# The Spectrum of Central Choriocapillaris Abnormalities on Swept-Source Optical Coherence Tomography Angiography in the Fellow Eye of Unilateral Exudative Age-Related Macular Degeneration Patients: From Flow Deficits to Subclinical Non-Exudative Neovascularization

**DOI:** 10.3390/jcm10122658

**Published:** 2021-06-16

**Authors:** Alexis Khorrami Kashi, Eric Souied, Selim Fares, Enrico Borrelli, Vittorio Capuano, Camille Jung, Giuseppe Querques, Alexandra Mouallem, Alexandra Miere

**Affiliations:** 1 Department of Ophthalmology, Centre Hospitalier Intercommunal de Créteil, University Paris Est Créteil, 94000 Créteil, France; alexis.khorrami@gmail.com (A.K.K.); eric.souied@chicreteil.fr (E.S.); selimfares8@gmail.com (S.F.); vittorio.capuano@gmail.com (V.C.); giuseppe.querques@hotmail.it (G.Q.); alexandra.mouallem@gmail.com (A.M.); 2 Clinical Research Center, GRC Macula, and Biological Ressources Center, Centre Hospitalier Intercommunal de Créteil, 94000 Créteil, France; camille.jung@chicreteil.fr; 3 Department of Ophthalmology, IRCCS San Raffaele Scientific Institute, University Vita-Salute San Raffaele, Via Olgettina 60, 20132 Milan, Italy; borrelli.enrico@yahoo.com

**Keywords:** AMD, oct-angiography, fellow eye, choriocapillaris, quiescent macular neovascularization

## Abstract

We evaluated the spectrum of choriocapillaris (CC) abnormalities in the fellow eyes of unilateral exudative age-related macular degeneration (AMD) patients using swept-source optical coherence tomography angiography (SS-OCTA). Fellow eyes of unilateral exudative AMD patients were prospectively included between May 2018 and October 2018. Patients underwent a multimodal imaging including a SS-OCTA. Demographics and clinical findings were analyzed. The estimated prevalence of macular neovascularization (MNV) was computed. Number and size of flow deficits (FDs) and percentage of flow deficits (FD%) were computed on the compensated CC flow images with the Fiji software. We included 97 eyes of 97 patients (mean age was 80 ± 7.66 years, 39 males, 58 females). The prevalence of MNV in the studied eyes was 8.25% (8/97 eyes). In the 89 non-neovascular eyes, FD% averaged 45.84% ± 11.63%, with a corresponding total area of FDs of 4.19 ± 1.12 mm^2^. There was a higher prevalence of drusenoid pigment epithelial detachment in eyes with subclinical neovascularization (*p* = 0.021). Fellow eyes with unilateral exudative AMD encompassed a series of CC abnormalities, from FDs of the aging CC to subclinical non-exudative MNV.

## 1. Introduction

Age-related macular degeneration (AMD) is a leading cause of vision loss in developed countries [1]. Among the hallmarks of AMD, drusen and pigment epithelial changes play a major part. Recently, Yu et al. have shown on the Age-Related Eye Disease Study Research Group 2 (AREDS2) eyes that, by 5 years, 67% of eyes with drusenoid pigment epithelial detachments (PED) progress to late a AMD, such as geographic atrophy (GA) or macular neovascularization (MNV) emanating from the choroid (type 1 and 2) or from the deep retinal layer (type 3) [2]. An increasing body of literature shows that besides inflammation or genetic predisposition, dysfunction of the complex comprised of retinal pigment epithelium (RPE)-Bruch’s membrane-choriocapillaris (CC) is mainly involved [3,4,5] in AMD pathogenesis and progression.

Choriocapillaris is a key source in the nutrition and oxygenation of the RPE. Bhutto and Lutty have shown that in atrophic AMD, the death and dysfunction of photoreceptors and CC appear to be secondary to the RPE loss, while in exudative AMD, the loss of choroidal vasculature may be the initial insult to the complex [6]. Loss of CC generates hypoxia in the RPE and consequently angiogenic substances are produced, abutting in the growth of neovascularization. Thus, the loss of CC might also be a stimulus for drusen formation because the disposal system for retinal debris and exocytosed material from RPE would be limited. Ultimately, the photoreceptors die of lack of nutrients, leakage of serum components from the neovascularization, and scar formation [6].

With the advent of optical coherence tomography angiography (OCTA), a depth-resolved non-invasive imaging technique, a detailed study of the CC in vivo has been possible [7,8]. Zheng et al. recently showed that, in normal aging eyes, the percentage of CC flow deficits (FDs) increases with age across the central 5 mm of the macula [9]. In intermediate AMD eyes, Borrelli et al. demonstrated an increased CC flow impairment, which co-localized to the area of CC beneath and immediately surrounding drusen [10]. Moreover, previous histological studies by Green and Sarks have described the presence of neovascularization that was not accompanied by exudation in postmortem eyes [11,12]. Querques et al. described this type of neovascularization in intermediate AMD eyes, coining the term of “quiescent” MNV by means of multimodal imaging accompanied by a functional characterization [13]. The detection of such quiescent neovascularization was possible by means of both indocyanine green angiography (ICGA) and spectral domain optical coherence tomography (SD-OCT), the latter revealing, at the site of quiescent MNV, an irregular, slightly elevated RPE with its major axis in the horizontal plane. Nonetheless, in recent years, OCTA has proven to be highly useful in detecting such quiescent neovascularization [14,15]. Therefore, given that subclinical, non-exudative MNV may precede the onset of exudation [16], our aim was to evaluate the spectrum of CC abnormalities in the fellow eyes of patients with unilateral exudative AMD using OCTA.

## 2. Materials and Methods

Consecutive patients with unilateral exudative AMD were enrolled in this prospective cross-sectional study at the Department of Ophthalmology at Créteil Hospital between May and October 2018. The institutional review boards of the University Paris-Est Créteil approved the study. The study was conducted in accordance with the tenets of the 1964 Declaration of Helsinki.

Fellow eyes of unilateral exudative AMD patients were included. Therefore, the fellow eye was considered as the study eye. Exclusion criteria consisted of active MNV, prior anti-vascular endothelial growth factor (anti-VEGF) treatment in the study eye, GA, and other confounding retinal disease, such as adult-onset foveomacular vitelliform dystrophy, high myopia, and diabetic retinopathy. Patients with a signal strength index <8 were also excluded.

Study eyes were classified as early AMD if drusen was <125 µm in diameter without any other abnormalities [17] present and as intermediate AMD if drusen was >125 µm with or without pigmentary abnormalities [17] present. Presence of small drusen (<63 µm), large drusen (>125 µm), drusenoid pigment epithelial detachment (PED) (>350 µm) and reticular pseudodrusen (RPD) were reported.

Subclinical non-exudative macular neovascularization (MNV) was defined as MNV without intraretinal/subretinal exudation on OCT but well detectable on fluorescein angiography (FA) and ICGA [13,14,15].

### 2.1. Imaging

All patients underwent a complete ophthalmological evaluation, including multimodal imaging: infrared imaging (IR), blue fundus autofluorescence (FAF), (Spectralis; Heidelberg Engineering, Heidelberg, Germany), spectral domain optical coherence tomography (Spectralis; Heidelberg Engineering, Heidelberg, Germany), and swept-source OCTA (Plex Elite, Carl Zeiss Meditec, Inc., Dublin, CA). All patients had had at least one FA and ICGA (Spectralis; Heidelberg Engineering, Heidelberg, Germany) previous to study inclusion.

Swept-source OCTA had an A-scan rate of 100,000 A-scans per second and a light source of 1050 nm (1000–1100 nm full bandwidth). The axial resolution was 6.3 µm and transverse resolution was 20 µm. We obtained 3 × 3 mm^2^ OCTA images centered on the fovea.

Grading was performed by expert readers on 3 × 3 mm^2^ SS-OCTA and corresponding structural OCT images, as well as on SD-OCT images and FAF. SS-OCTA and structural SS-OCT images were independently analyzed by two expert readers (SF and AMB) to confirm the presence/absence of drusen, subclinical non-exudative macular neovascularization, or macular atrophy. In case of disagreement, a third reader (AM) adjudicated discordances. SD-OCT images were analyzed by two expert readers (AKK and VC) to confirm presence and type of drusen and to measure central macular thickness (CMT) and choroidal thickness (CT). In case of disagreement, a third reader (ES) adjudicated discordances. Presence/absence of atrophy was assessed on FAF images (CJ, AM). CMT was recorded from the retinal thickness ETDRS grid generated by the Spectralis software (Version 1.10.4.0, Heidelberg Engineering, Heidelberg, Germany). CT was determined using a scan passing through the central fovea and defined as the distance between the retinal pigment epithelium–Bruch’s membrane complex and the sclerochoroidal interface [18]. Calipers provided by the OCT software were used for this measurement.

### 2.2. Image Processing

En face CC structure and en face CC flow images were extracted from the OCTA device. Manual segmentation was used to segment the CC slab as previously described [10] (10 µm thick segments, starting 31 µm posterior to the RPE fit reference). Segmentation errors, if present, were manually adjusted by one reader (AM) in order to correct segmentation. Structural and vascular images were then processed.

**Figure 1:**
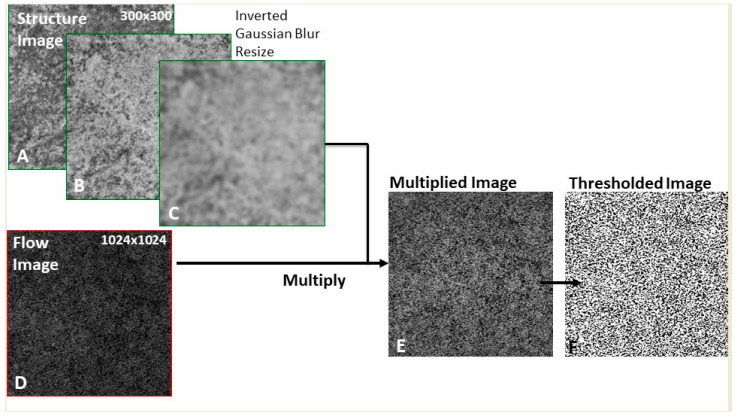
Representation of the algorithm used to investigate the choriocapillaris (CC). To the en face CC structure image (**A**) an inverse transformation was applied using the Fiji “Invert” function (**B**). Smoothing was obtained using the Gaussian blur filter (**C**). Multiplication between the en face CC flow image (**D**) and the processed en face CC structure image (**C**) was performed using “Image Calculator”. A compensated en face CC image was obtained (**E**). Binarization of the compensated en face CC flow image was performed using the Phansalkar threshold (radius: 15 pixels) in order to obtain a quantitative analysis of the flow deficits as described in recent literature (**F**).

Images were then imported into the Fiji Software (National Institute of Mental Health, Bethesda, MD, USA). In order to compensate the shadowing generated by drusen onto the CC, we used a previously described method of compensation for the signal attenuation under drusen [10,19] (Figure 1).

En face CC flow images were compensated with en face CC structures as follows: To an en face CC structure image, an inverse transformation was applied using the Fiji “Invert” function. Gaussian blur filter was applied on the en face CC structure image for smoothing. Multiplication between the en face CC flow image and the processed en face CC structure image was performed using “Image Calculator”. Thus, we obtained a compensated en face CC image. Binarization of the compensated en face CC flow image was performed using the Phansalkar threshold (radius: 15 pixels) in order to obtain a quantitative analysis of the flow deficits as described in recent literature [10]. The percentage of flow deficits (FD%), number and size of flow deficits (FDs), the total FDs area were obtained using the “Analyze Particles” module (size: 0–infinity, circularity: 0–1) [10].

The number of FDs was the number of contiguous black pixel areas representing the flow deficits. The size of FDs was the mean area, in µm^2^, of contiguous black pixel area. The total FDs area represents the area, in mm^2^, of all the FDs. The FD% was computed as the total FDs area on the total image (black and white pixels) area.

### 2.3. Statistical Analysis

Qualitative variables were expressed in percentages and quantitative variables were expressed by their mean with standard deviation. Comparisons of qualitative variables were performed using the Fischer exact test. Comparisons of quantitative variables were performed using the Mann–Whitney test. We tested and compared different radii within the compensation algorithm [20]: radius of 4 pixels, corresponding to 26.37 microns, radius of 8 pixels (49.80 microns), 10 pixels (61.52 microns), and 15 pixels (90.82 microns). The coefficient regression values were calculated using the linear regression method. Multivariate analyses were performed to search factors associated with FD%, size and number of FDs expressed as log10. They took into account the following parameters: age, best corrected visual acuity (BCVA, in logMAR), CMT, CT (in log10), small drusen, large drusen, drusenoid PED, RPD, and atrophy that were associated in univariate analysis (*p* < 0.20) and/or clinical significance. A relation between the FD size and FD number was assessed by using the logarithm of the FD size and FD number values and by carrying out a linear regression [7]. *p* < 0.05 was retained as significant. Analyses were performed with STATA, version 13.0. (Texas, USA).

## 3. Results

### 3.1. Patient Demographics and Clinical Characteristics

We included 97 eyes of 97 patients (mean age was 80 ± 7.66 years, 39 males, 58 females). In our series, unilateral exudative AMD consisted of type 1 MNV in 61/97 eyes, type 2 MNV in 15/97 eyes, type 3 MNV in 8/97 eyes and mixed type 1 and 2 in 13/97 eyes.

The fellow eye was considered as the study eye. In our series, 62/97 study eyes presented with small drusen, 69/97 with large drusen, 44/97 with drusenoid PED, 54/97 with reticular pseudodrusen (RPD), while 19/97 presented with macular atrophy on FAF imaging and SD-OCT. In all the eyes, macular atrophy was present outside the 10 central degrees analyzed.

On the overall cohort, mean CMT was 238.49 µm ± 67.49 µm and mean CT was 210.51 µm ± 83.21 µm.

### 3.2. OCTA Spectrum of Choriocapillaris Abnormalities

Mean PlexElite Image Quality was 8.99 (±0.74).

#### 3.2.1. Neovascularization: Subclinical Non-Exudative MNV

Table 1 summarizes the characteristics of the neovascular study eyes (*n* = 8).

**Figure 2:**
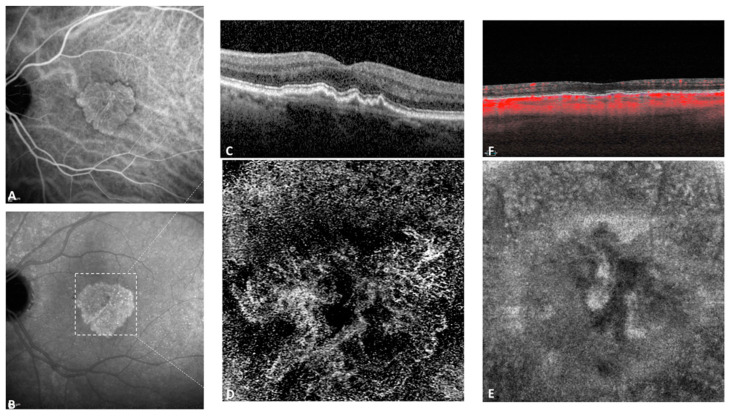
Multimodal imaging of an 80 years old patient with subclinical macular non-exudative neovascularization on the study eye. Intermediate (**A**) and late (**B**) indocyanine green angiography (ICGA) frames, revealing a hyperfluorescent plaque in the late frame. Spectral domain optical coherence tomography (SD-OCT) (**C**) showed a shallow pigment epithelial detachment (PED). The outer retina to choriocapillaris (ORCC) segmentation of the 3 × 3 mm^2^ OCTA (**D**) revealed the presence of a high flow neovascular network. (**E**). Corresponding structural en face image at the choriocapillaris and (**F**) Corresponding B-scan with the choriocapillaris segmentation.

**Figure 3:**
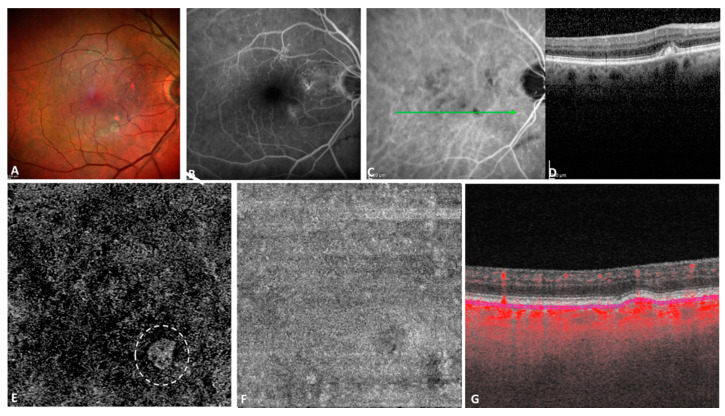
Multimodal imaging of an 86 years old patient with a small subclinical macular non-exudative neovascularization on the study eye. Multicolor imaging revealing soft drusen (**A**) that appeared hyperfluorescent on fluorescein angiography (FA) (**B**) and hypofluorescent on the late indocyanine green angiography (ICGA) frame. (**D**) Spectral domain optical coherence tomography (SD-OCT) revealed a small hyperreflective drusen, corresponding to a hypofluorescent area on ICGA (**C**). The manual 10 µm thick segmentation located 31 μm posterior to the RPE fit reference with projection artifact removal (**E**), revealed the presence of a small high flow neovascular network (dashed white circle) with the en face structural slab (**F**). The corresponding B-scan with flow overlay (**G**) confirmed the presence of flow within the drusen.

Of the 97 study eyes, OCTA detected the presence of neovascularization in 8 (Figure 2 and Figure 3), thus the estimated prevalence was 8.25%. Table 2 shows the comparison between the non-neovascular and the neovascular study eyes. None of the 8 neovascular eyes presented with macular atrophy. However, 3/8 of these presented with small drusen, 4/8 with large drusen, 7/8 with drusenoid PED, and 4/8 with RPD. Mean CMT in these eyes was 250.13 µm (±40.95) and mean CT was 227 µm (±94.38).

There were no statistically significant differences between the non-neovascular (*n* = 89) and the neovascular (*n* = 8) eyes in terms of quantitative and qualitative variables except for the presence of drusenoid PED (*p* = 0.021) (Table 2).

#### 3.2.2. Choriocapillaris Flow Deficits in Non-Neovascular Fellow Eyes

**Figure 4:**
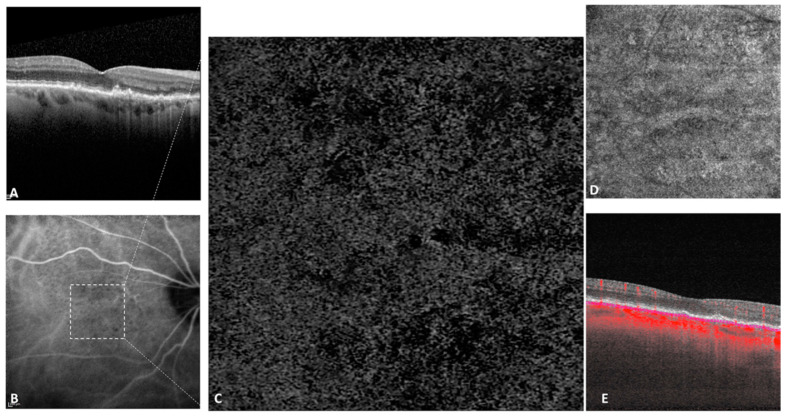
Choriocapillaris abnormalities of an eye with intermediate AMD. Enhanced depth imaging spectral domain optical coherence tomography (EDI-SD-OCT) visualized numerous drusen (**A**) which appeared hypofluorescent on indocyanine green angiography (ICGA) (**B**). On the manual choriocapillaris segmentation of the 3 × 3 mm^2^ OCTA (**C**), multiple flow deficits were noticed in the CC flow image, corresponding in part to signal attenuation generated by drusen. Panel (**D**) corresponds to the structural en face CC slab, while panel (**E**) represents the corresponding B-scan with flow overlay.

**Figure 5:**
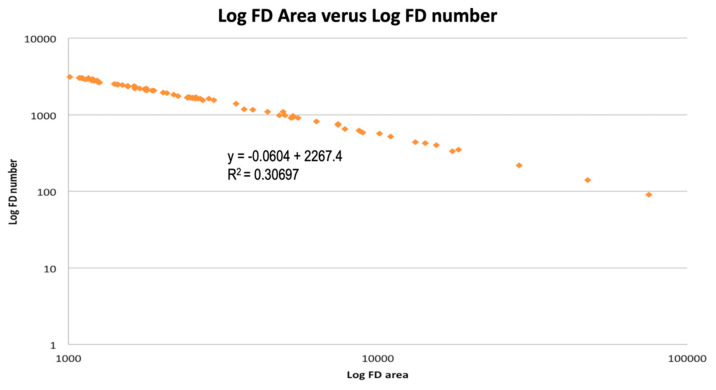
Log–log graph expressed in log10 between the size and number of flow deficits. When the log flow deficits were plotted against the log of the size of the flow deficits, a linear relationship was observed, with an intercept of +2267.4 and a slope of −0.0604.

Concerning the CC abnormalities of non-neovascular fellow eyes (*n* = 89), FD% averaged 45.84% (±11.63). Figure 4 illustrates the CC FD in a non-neovascular fellow eye. The mean number of FDs was 1965 (±1063) and the mean size of FDs was 5076.49 µm^2^ (±9984.51). Figure 5 illustrates the log–log graph between FD size and FD number with an intercept of +2267 and a slope of −0.0604, showing an inverse linear relation. The mean area of FDs was 4.19 (±1.12) mm^2^. Table 3 summarizes the mean values of CC abnormalities.

In the univariate analysis, FD% in the non-neovascular fellow eyes was not statistically associated with CMT (*p* = 0.59), small drusen (*p* = 0.55), nor drusenoid PED (*p* = 0.09), but it was statistically associated with CT (*p* = 0.003), large drusen (*p* = 0.03), atrophy (*p* = 0.02), and RPD (*p* = 0.007). These associations were not found in the multivariate analysis, where FD% was associated in a statistically significant manner with age (linear regression, coefficient 0.62, *p* = 0.02) and the presence of small drusen (linear regression, coefficient 0.13, *p* = 0.017).

The size of FDs was not correlated with variables such as CMT (*p* = 0.64), small drusen (*p* = 0.49), large drusen (*p* = 0.07), and drusenoid PED (*p* = 0.13) in the univariate analysis, where it was associated with atrophy (*p* = 0.029) and RPD (*p* = 0.02). These associations were not found in the multivariate analysis. However, age (linear regression, coefficient 2.69, *p* = 0.019) and choroidal thickness (linear regression, coefficient −0.68, *p* = 0.005) were associated, in the multivariate analysis, with the size of FDs.

The number of FDs was not correlated with variables such as CMT (*p* = 0.59), small drusen (*p* = 0.76), large drusen (*p* = 0.19), nor drusenoid PED (*p* = 0.21) in the univariate analysis. However, the number of FDs was correlated with findings such as atrophy (*p* = 0.03) and RPD (*p* = 0.048) on multimodal imaging. These correlations were not found in the multivariate analysis, where the number of FDs was associated with age (coefficient −2.25, *p* = 0.007) and CT (coefficient 0.46, *p* = 0.008). Table 4 summarizes these findings.

#### 3.2.3. Sensitivity Analysis

**Figure 6:**
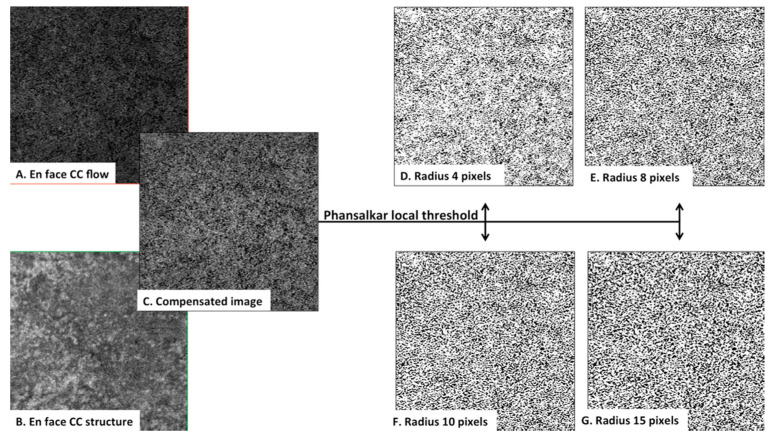
Phansalkar’s local thresholding method with window radii of 4, 8, 10, and 15 pixels on a 3 × 3 mm^2^ swept source optical coherence tomography angiography (SS-OCTA) scan from an intermediate AMD eye. Flow choriocapillaris (CC) SS-OCTA image (**A**) and the en face CC structure slab (**B**) generated the compensated image (**C**). Panels (**D**) to (**G**) show the effect of choosing different window radii ((**D**) radius 4 pixels, (**E**) radius 8 pixels, (**F**) radius 10 pixels, (**G**) radius 15 pixels)in Phansalkar’s local thresholding method for 3 × 3 mm SS-OCTA images.

We tested the compensation algorithm for different radii values—radius of 4 pixels, corresponding to 26.37 microns, radius of 8 pixels (49.80 microns), 10 pixels (61.52 microns) and 15 pixels (90.82 microns) (Figure 6)—and the results are summarized in Table 5. According to the different radii, FD% ranged from 41.74 to 45.84%, mean number of FD from 3381.08 to 1964.74, and mean size of FD from 4198.77 to 5548.82 µm^2^.

## 4. Discussion

In our study, we showed that in the fellow eye of exudative AMD patients, there is a wide spectrum of macular choriocapillaris abnormalities, ranging from subclinical non-exudative neovascularization to an increase in CC FD%.

Measurable changes of the flow within the CC were found with age and hypertension, and significant alterations in the flow pattern were demonstrated in eyes with RPD and in eyes with late AMD in the fellow eye [3,7,9,10,21,22,23,24].

Zheng et al. recently showed that in normal eyes, there was an increasing number and percentage of CC FDs with ageing [9]. Thus, the mean FD% in normal eyes within the age category corresponding to our cohort (80–89 years) was 17.0% ± 3.3% on the 2.5 mm circle on the 3 × 3 mm^2^ slab, while in our study, the mean percentage of CC FD% in our non-neovascular, mostly intermediate AMD (iAMD) group (*n* = 89) was 45.84% (±11.63) on the whole image.

Furthermore, Sacconi et al. performed CC perfusion density (PD) in healthy subjects using SS-OCTA and a similar image processing method [21]. The authors found a significant negative association between perfusion of CC and age in healthy subjects. In 12 eyes of 70–80 years old healthy patients, a mean PD of 71.791% ± 4.113% on the 3 mm annulus around the fovea was reported, accounting for a CC FD% of 28.209%. Nevertheless, there is a significant difference between these results of PD and/or FD% and those of Zheng et al. [9]. These differences may be explained by the fact that small variations in image processing (thresholding, used radius, slab selection) can greatly influence the results [25,26]. Hence, these considerations should be accounted for the discrepancies between our study and previous studies. Moreover, Chu et al. recently demonstrated that the local window radius should be optimized before using the Phansalkar’s thresholding method [20]. Therefore, in the present study, radius 15 was chosen for the main analysis, in accordance with recent literature. However, a supplementary sensitivity analysis with different radii values (4, 8, and 10) was also performed (Table 5 and Figure 6), showing up to 4.10% difference for FD%. As too small radii may not include enough pixels and larger radii generate larger FD% values, a consensus on the parameters of choriocapillaris analysis is needed. Slight differences in manual segmentation and image processing methods lead to an important variation in the CC quantitative variables. In the present study, we tried to overcome this variability by using the same image processing method as other SS-OCTA studies [10] and testing different radii.

Borrelli et al. quantitatively analyzed the CC signal voids’ topographical distribution in single images of iAMD eyes [10], showing consistent results with those found among our (non-neovascular) iAMD eyes. In an analysis comparing the entire CC area in both control eyes and the drusen-free region in iAMD, the multiple regression analysis showed that only age was associated with the CC variables. In our study, we performed a linear regression on FD%, size of FD, and number of FD showing, in the multivariate analysis, a statistically significant association with age for each of the CC variables studied. Moreover, FD size and FD number demonstrated an inverse linear relationship on the log–log plot in Figure 5, which is consistent with recent literature [7]. Additionally, in the multivariate analysis, small drusen was also associated with FD%, while CT was associated with the size and number of FD (Table 4).

Nassisi et al. recently showed that there is a correlation between the CC flow impairment around the atrophic lesions and the yearly growth rate of GA, with more CC flow impairment meaning a faster growth of atrophy [27]. In our study, the presence of atrophy was associated, in the univariate analysis, with FD%, FD number and size, suggesting that the increase of FD% may indeed correlate with atrophy development.

In our study, subclinical non-exudative neovascularization was present in 8 out of 97 eyes, suggesting an estimated prevalence of neovascular complications of 8.25% in the fellow eye of exudative AMD. Interestingly, Treister et al. showed a slightly higher prevalence of subclinical neovascularization (14.7%) in the 34 fellow eyes of unilateral late AMD patients, analyzed by SD-OCTA [28].

On one hand, de Oliveira Dias et al. have recently shown, by means of SS-OCTA, a prevalence of 14.4% of subclinical non-exudative neovascularization among the 160 eyes included in their cohort [29]. Recently, Yang et al. [16] found, also by means of SS-OCTA, that 13.2% of their cohort (non-exudative AMD, either iAMD or late AMD) had subclinical MNV at first imaging.

The use of a small scanning area, as well as the lack of subfield analysis, are among the limitations of our study, together with the small sample size and the absence of a control group. The absence of a control group that would have underwent the same image processing method is the reason why we cannot conclude, despite the comparison with other studies, that the observed changes in the CC were due to iAMD rather than to other factors. Moreover, the CC slab in eyes with reticular pseudodrusen and decreased choroidal thickness might generate the displacement of the slab from the CC to the anterior choroid. It remains difficult to select a cutoff of choroidal thickness from which this slab should be adapted. The manual segmentation was manually corrected if necessary. Nonetheless, choosing a 3 × 3 mm^2^ scanning area allowed us to use the highest resolution scanning area in order to assess CC FDs.

One of the strengths of the present study was the use of the SS technology, which allowed for a better detection of MNV and a more reliable assessment of CC nonperfusion.

In conclusion, fellow eyes of unilateral exudative AMD encompass a series of CC abnormalities, from FDs of the ageing CC to subclinical non-exudative MNV.

## Figures and Tables

**Table 1:** Demographics, clinical findings, and multimodal imaging features of patients (*n* = 8) with subclinical non-exudative macular neovascularization on the study eye.

Patient	Gender	Age	BCVA (logMAR)	BCVA (Snellen Equivalent)	Small Drusen	Large Drusen	Drusenoid PED	Atrophy	Reticular Pseudodrusen	CMT (µm)	CT (µm)
1	Female	72	0.1	20/25	1	1	1	0	1	252	257
2	Female	78	0.0	20/20	0	0	1	0	0	211	139
3	Male	80	0.2	20/32	0	0	1	0	0	338	136
4	Male	82	0.1	20/25	0	0	0	0	0	249	289
5	Female	91	0.3	20/40	1	1	1	0	1	264	243
6	Female	72	0.1	20/25	1	1	1	0	1	244	382
7	Female	89	0.2	20/32	0	0	1	0	1	239	268
8	Female	88	0.0	20/20	0	1	1	0	0	204	102

BCVA—best-corrected visual acuity; PED—pigment epithelial detachment; CMT—central macular thickness; CT—choroidal thickness; 0—absence; 1—presence.

**Table 2:** Comparison between the non-neovascular (*n* = 89) and the neovascular (*n* = 8) study eyes.

	Non-Neovascular Study Eyes (*n* = 89)	Neovascular ^†^ Study Eyes (*n* = 8)	*p*-Value
Mean Age (years)	79 (±7.70)	82 (±7.41)	0.41 *
BCVA (logMAR)	0.18 (±0.46)	0.12 (±0.10)	0.21 *
Small drusen (*n*,%)	59 (66.29%)	3 (37.5%)	0.13 **
Large drusen (*n*,%)	65 (73.03%)	4 (50%)	0.22 **
Drusenoid PED (*n*,%)	37 (41.57%)	7 (87.50%)	0.021 **
Reticular pseudodrusen (*n*,%)	50 (56.18%)	4 (50%)	1.0 **
Atrophy (*n*,%)	19 (21.35%)	0 (0%)	0.34 **
CMT (µm)	231.95 (±43.74)	250.13 (±40.95)	0.20 *
CT (µm)	209.09 (±83.04)	227 (±94.38)	0.63 *

†—subclinical non-exudative macular neovascularization; *—Mann–Whitney Test; **—Fischer’s exact test; BCVA—best-corrected visual acuity; PED—pigment epithelial detachment; CMT—central macular thickness; CT—choroidal thickness.

**Table 3:** Choriocapillaris abnormalities in the non-neovascular group (*n* = 89) using radius 15 pixels.

	Mean (SD)
**Percentage of flow deficits (%)**	45.84 (±11.63)
**Number of flow deficits**	1964.74 (±1063.19)
**Size of flow deficits (µm^2^)**	5076.49 µm^2^ (±9984.51)
**Area of flow deficits (mm^2^)**	4.19 (±1.12)

**Table 4:** Linear regression on percentage of flow deficits (FD), size of FD, number of FD in non-neovascular study eyes.

Linear Regression on	Variable	Univariate Analysis			Multivariate Analysis		
		Coef. ^†^	Std. Err.	*p* Value	Coef. ^†^	Std. Err.	*p* Value
Percentage of flow deficits *	Age *	0.77	0.26	0.004	0.62	0.26	0.02
	CMT *	−0.04	0.07	0.59			
	CT *	−0.16	0.05	0.003			
	Small drusen	0.03	0.05	0.55	−0.13	0.05	0.017
	Large drusen	0.12	0.06	0.03			
	Drusenoid PED	0.09	0.05	0.09			
	Atrophy	0.14	0.06	0.02			
	RPD	0.14	0.05	0.007			
Size of flow deficits *	Age *	3.44	1.13	0.003	2.69	1.13	0.019
	CMT *	−0.15	0.32	0.64			
	CT *	−0.81	0.23	0.001	−0.68	0.23	0.005
	Small drusen	0.16	0.24	0.49			
	Large drusen	0.47	0.25	0.07			
	Drusenoid PED	0.34	0.23	0.13			
	Atrophy	0.61	0.27	0.029			
	RPD	0.53	0.22	0.02			
Number of flow deficits *	Age *	−2.74	0.80	0.001	−2.25	0.80	0.007
	CMT *	0.12	0.23	0.59			
	CT *	0.57	0.17	0.001	0.46	0.17	0.008
	Small drusen	−0.05	0.17	0.76			
	Large drusen	−0.24	0.18	0.19			
	Drusenoid PED	−0.21	0.16	0.21			
	Atrophy	−0.43	0.19	0.03			
	RPD	−0.33	0.16	0.048			

*—expressed in log10; ^†^- regression coefficient; CMT—central macular thickness; CT—choroidal thickness; PED—pigment epithelial detachment; RPD—reticular pseudodrusen.

**Table 5:** Choriocapillaris abnormalities in the non-neovascular group according to radii (*n* = 89).

	Mean (SD) Radius 4	Mean (SD) Radius 8	Mean (SD) Radius 10	Mean (SD) Radius 15 Pixel
Percentage of flow deficits (%)	41.74 (±15.26)	44.81 (±12.43)	45.52 (±12.01)	45.84 (±11.63)
Number of flow deficits	3381.08 (±1735.01)	2405.87 (±1347.19)	2151.26 (±1218.96)	1964.74 (±1063.19)
Size of flow deficits (µm^2^)	4198.77 (±14,929.13)	5253.17 (±13,861.42)	5548.82 (±13,471.32)	5076.49 (±9984.51)

## Data Availability

Data are available from the corresponding author upon reasonable request.
